# Effect of nano-micelle curcumin on hepatic enzymes: A new treatment approach for non-alcoholic fatty liver disease (NAFLD)

**DOI:** 10.22038/AJP.2023.21919

**Published:** 2023

**Authors:** Ali Beheshti Namdar, Mitra Ahadi, Seyed Mousalreza Hoseini, Hassan Vosoghinia, Hosein Rajablou, Salman Farsi, Amirsadra Zangouei, Hamid Reza Rahimi

**Affiliations:** 1 *Department of Internal Medicine, Faculty of Medicine, Mashhad University of Medical Sciences, Mashhad, Iran*; 2 *Student Research Committee, Faculty of Medicine, Mashhad University of Medical Sciences, Mashhad, Iran *; 3 *Department of Medical Genetics & Molecular Medicine, Faculty of Medicine, Mashhad University of Medical Sciences, Mashhad, Iran*; 4 *Vascular and Endovascular Surgery Research Center, Mashhad University of Medical Sciences, Mashhad, Iran*

**Keywords:** Non-alcoholic fatty liver disease, Curcumin, Nano-micelle, hepatic enzymes

## Abstract

**Objective:**

Nonalcoholic fatty liver disease (NAFLD) is characterized by excessive lipid accumulation in hepatocytes with no consumption of alcohol. Recently, curcumin is a natural polyphenol found in turmeric has been examined for the treatment of NAFLD. This study aimed to assess the efficacy of 160 mg/day nano-micelle curcumin on the amelioration of NAFLD by measuring liver enzymes.

**Materials and Methods:**

Patients with NAFLD were randomly divided into curcumin (intervention group n=33) and placebo (n=33) groups and at the end of the study, the data of 56 participants who completed the 2-month intervention were analyzed. Laboratory tests and questionnaires were used to gather information. Both groups received recommendations for lifestyle modification, and were advised to other necessary advices. Patients in the curcumin group received 160 mg/day of nano-micelle curcumin in two divided doses for 60 days. The 2 groups were followed up for two months and clinical and laboratory indices were compared.

**Results:**

Our data showed a significant decrease in alanine aminotransferase (ALT) and aspartate aminotransferase (AST) in the curcumin group (p<0.01) as well as a significant difference between the groups before and after the intervention in curcumin group (p<0.05). Interestingly, a meaningful decrease in AST serum level was observed in the intervention group (p<0.01).

**Conclusion:**

Our study demonstrated that short-term supplementation with nano-micelle curcumin results in the reduction of AST and ALT and is beneficial for the treatment of NAFLD.

## Introduction

Nonalcoholic fatty liver disease (NAFLD) is an umbrella term including a large variety of liver diseases that despite mimicking alcohol-induced liver diseases (Serfaty and Lemoine, 2008 ▶), are not caused by alcohol consumption (Lim et al., 2015 ▶) and range from steatosis, usually a benign and non-progressive condition, to non-alcoholic steatohepatitis (NASH), which may lead to fibrosis, cirrhosis and other complication like hepatic carcinoma (Spooner and Jump, 2019 ▶; Sutti and Albano, 2020 ▶). It is histologically characterized by fat accumulation in at least 5% of hepatocytes (Tiniakos et al., 2010 ▶). NAFLD is a major health concern worldwide (Rahmani et al., 2016 ▶). It is the most prevalent liver disease in western countries affecting 27-34% of the population (Almobarak et al., 2014 ▶; Fazel et al., 2016 ▶), and its prevalence in Asia is estimated to be 10-30% (Kim et al., 2014 ▶). Type 2 diabetes mellitus and obesity are two important risk factors for developing NAFLD; studies have shown that 76% of type 2 diabetics (Chalasani et al., 2012 ▶) and 96% of obese undergoing bariatric surgeries (Lam and Younossi, 2010 ▶) suffer from NAFLD.

 NAFLD diagnosis contains histological tests, ultrasonography and laboratory enzymatic measurements consisting of alanine aminotransferase (ALT), aspartate transaminase (AST), and γ-Glutamyl transpeptidase (GGT) in order to evaluate liver functions (Festi et al., 2013 ▶). There are many different drugs (such as metformin, pioglitazone, and vitamin E) proposed for the treatment of NAFLD, yet there is no certain treatment for it (Slika and Patra, 2020 ▶); diet and lifestyle modifications have been shown to be effective approaches (Weiß et al., 2014 ▶). 

Curcumin is under evaluation to determine if it is beneficial in the treatment of NAFLD. Curcumin is a polyphenolic pigment obtained from turmeric (Slika and Patra, 2020 ▶). Studies have reported the anticarcinogenic, anti-hyperlipidemic, anti-inflammatory, antioxidant, antidepressant, antimicrobial, and anti-arthritic effects of curcumin (Hosseini et al., 2018 ▶, Jalali et al., 2019 ▶; Shahcheraghi et al., 2020 ▶). 

Oxidative stress exerts a key role in the development and progression of NAFLD (Rahimi et al., 2015 ▶; Mokgalaboni et al., 2021 ▶). As shown in several experimental and clinical trials, curcumin reduces predominant markers of oxidative stress and suppresses inflammatory pathways such as NF-κB (Rahimi et al., 2016 ▶; Bateni et al., 2021 ▶; Mokgalaboni et al., 2021 ▶). There is scarce information about the role of curcumin in nano-micelle form in the treatment of NAFLD. With regard to curcumin bioavailability, curcumin dosage is an important issue in NAFLD treatment to find the optimum dose. To date, no research has been conducted using 160 mg/day curcumin in form of nanoparticles. Previous similar studies utilized phytosomal (Panahi et al., 2016 ▶; Panahi et al., 2017 ▶) and amorphous dispersion curcumin (Rahmani et al., 2016 ▶) which are impure; therefore, the results may not be accurate because the gross substance may cause an interruption in the final result of the unknown behavior in bioavailability of pure or other form of Curcumin. 

Moreover, curcumin encapsulated in form of nano-micelle has been shown to be 100% pure (Rahimi et al., 2016 ▶), which may lead to a much better outcome. According to a pervious study at Mashhad University of Medical Sciences, it was indicated that nano-micelles (SinaCurcumin^®^ by Exir Nano Sina Company) could improve solubility, oral bioavailability, and the stability of curcuminoids (Hatamipour et al., 2019 ▶). 

Considering the histologic structure of the liver and long-term prognosis, NAFLD has 3 grades (Mahaling et al., 2013 ▶). This classification does not correlate with liver function tests and liver enzymes. AST and ALT levels are not good indicators to determine the stage of fibrosis and steatosis in NAFLD patients (Khodadoostan et al., 2016 ▶). According to Jazayeri-Tehrani et al. nano-curcumin could improve glucose and lipid indices and reduce inflammation (Jazayeri-Tehrani et al., 2019 ▶), so nanoformulation of curcumin may increase bioavailability and decrease fat tissue inflammation in NAFLD patients (Varì et al., 2021 ▶).

In this study, we aimed to examine whether 160 mg/day curcumin supplementation delivered in form of nano-micelle, has a considerable effect on the disease by measuring the liver enzymes in NAFLD patients. 

## Materials and Methods

**Study design and participants**

A double-blind randomized clinical trial (registration code: IRCT2017012031423N1) was conducted at the special clinic of Ghaem Academic Hospital affiliated with Mashhad University of Medical Sciences in 2017. 56 hepatosteatosis (fatty liver) patients aged 15 to 60 years old (36 male and 20 female finished the intervention) were randomly assigned to two groups: 1) 160 mg/day nano-micelle curcumin divided into 2 doses (SinaCurcumin^® ^Exir Nano, Tehran, Iran) for 60 days (Hatamipour et al., 2019 ▶), and 2) placebo for 60 days (produced by Exir Nano, Tehran, Iran). All patients were observed and followed for 2 months by the same researcher during the study, and in case any patients had acute drug reactions, they could report it through a direct phone hotline.

**Randomization and blinding procedures**

**Figure 1:**
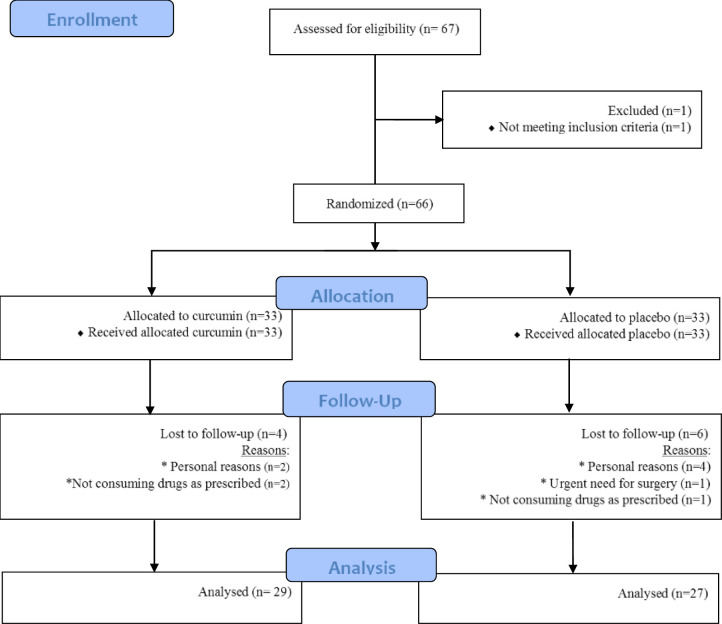
Flowchart of the trial

Patients were divided into curcumin and placebo groups by specialist based on random digits table. Patients with even numbers received nano-micelle curcumin and those with odd numbers received placebo. Limitations of calorie intake and training were recommended to both groups; this study was managed with per-protocol analysis, and those who did not complete their interventions were excluded (Figure 1. shows the flowchart of the trial). It should be noted that the study was double-blinded and the placebo and curcumin drug codes were unclear to the patient and the researcher until the end of the design. 

**Inclusion and exclusion criteria**

Participants whose ALT and AST levels were 1.5 times higher the normal range and had diabetes, dyslipidemia, or obesity were eligible to enter the study. Dyslipidemia was defined as having inappropriate higher amounts of lipids or fat in the bloodstream including hypertriglyceridemia, hypercholesterolemia and lower-than-normal levels of high-density lipoprotein (Chobanian et al., 2003 ▶). A participant with a fasting blood glucose level of 126 mg/dl or higher was considered to have diabetes (Association, 2009 ▶).

 Individuals who had history of alcohol use, pregnancy, positive serological tests for hepatitis B and C, upper than 450 ferritin level, iron/total iron-binding capacity (TIBC) higher than 45%, positive anti-nuclear antibody (ANA) or antimitochondrial antibodies (AMA) test, low level of ceruloplasmin, high levels of anti-smooth-muscle antibody (ASMA) and liver kidney microsome type 1 (LKMI) antibodies and developed a chronic or an acute illness (increasing erythrocyte sedimentation rate (ESR) or C-reactive protein (CRP) in the study time), rheumatic disease, renal disease, any type of cancer, infectious disease in the last 3 months, people who had undergone any surgical procedures in the preceding 3 months, subjects who had a history of angioplasty or coronary by-pass graft surgery, subjects who had a traumatic or major immunologic diseases, subjects who were using steroids, penicillin, oral contraceptive or hormone replacement therapy or any of other medications which were defined previously (Björnsson, 2016 ▶; Rahimi et al., 2016 ▶), or subjects with any inflammatory disease such as inflammatory bowel disease, psoriasis, multiple sclerosis, systemic lupus erythematosus, myasthenia gravis, or autoimmune thyroiditis were excluded from the study. 

**Collecting subjects' data**

A questionnaire was utilized to gather information such as demographic data and anthropometric parameters. Height was determined by a wall-mounted stadiometer. Weight was measured using an electronic scale while the subject was wearing light clothing without shoes. During the two-month intervention, patients were followed up weekly; all patients were also examined by the same sonographer and ultrasound device to detect the presence of fatty liver. The checklists were then filled out with the supervision of our team researchers and the patients were referred to the coordinated laboratory for required examinations.

**Laboratory tests**

Serum enzymatic assay (alanine transaminase (ALT), aspartate transaminase (AST), and alkaline phosphatase (ALP) and serum bilirubin assay (total and direct) were prepared by 3000 BT equipment. Blood triglyceride, cholesterol, high-density lipoprotein, low-density lipoprotein, fasting blood sugar, and insulin were measured as reported previously (Panahi et al., 2016 ▶).

**Ethical issues**

Before entering the study, all participants were informed about the aim of the study; verbal and written consent were also obtained. All subjects agreed to the study conditions. Those who preferred to decline at any stage were withdrawn from the research. Furthermore, we had a hotline for reporting any acute drug reactions or other complications. This research was approved by the Ethics Committee of Mashhad University of Medical Sciences (registration code: IRCT2017012031423N1). 

**Statistical analysis**

The sampling method was based on inclusion and exclusion criteria, but we used random digits table in order to divide subjects into the two groups. According to previous similar researches (Chuengsamarn et al., 2014 ▶; Rahmani et al., 2016 ▶; Panahi et al., 2017 ▶), the minimum sample size for each group was determined 25 with an 80% power and 5% level of significance by statistical specialist according to Panahi et al. (Panahi et al., 2016 ▶). Data were analyzed using SPSS for Windows™, version 16 software package (SPSS Inc., Chicago, IL, USA). 

Firstly, the assessment of the data with the Kolmogorov-Smirnov test showed that they were normally distributed, so they are expressed as Mean±SD. Comparison of the groups was made with the paired t-test. A two-sided p-value <0.05 was considered statistically significant.

## Results

**Baseline characteristics**

Our data showed that whole characteristics had normal distribution by Kolmogorov-Smirnov test. Furthermore, sex distribution in the curcumin and placebo groups were matched by the chi-square test (p>0.05). Regarding the ultrasonographic findings, there was no significant difference between the groups. 

Before the intervention, the age of participants was significantly different between the two groups (p<0.05), however, others baseline demographic and biochemical serum factors were matched (Table 1).

**Enzymatic assay**

**Figure 2:**
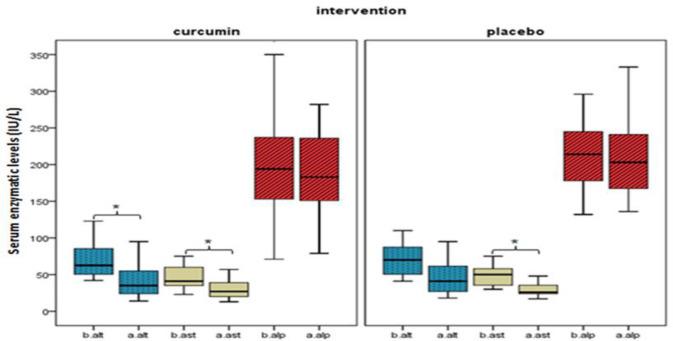
Effect of curcumin and placebo intervention on hepatic enzymes. b. and a. mean serum enzymatic activity before(b.) and after(a.) the intervention. *p-value<0.01 denotes significant difference between before and after the trial

The results of the associational analysis are shown in Table 2. In the curcumin group, AST and ALT enzymes showed a significant decrease (p<0.01), but no such significant reduction was observed for ALP enzyme. The most interesting aspect of this Table is that AST enzyme showed meaningful association different in the placebo group (p<0.01). Figure 2 compares the summary statistic before and after the intervention in both groups. In addition, we investigated the relation between further variables (anthropometric, demographic and biochemical) and curcumin intervention after 8 weeks. Although no significant association was found in all other variables, triglyceride (TG), total cholesterol (CHOL), and low density lipoprotein (LDL) had an observable decrease.

**Table 1:** Overall characteristics of the study population before intervention (Mean±SD)/ (Median (IQR))

**Variables **			**Groups**	
			**Curcumin** ** N=27**	**Placebo** **N=29**
**Dependent**	ALT (IU/L)		73.27±29.70	74.25±29.74
	AST (IU/L)		49.93±22.73	50.37±17.47
	ALP (IU/L)		203.10±68.52	212.66±46.03
**Independent **	Anthropometric	BMI (kg/m^2^)	29.90±4.58	29.68±4.61
	Sex	Male (No/%)	21(78%)	23(79%)
	Demographic	Weight(kg)	82.34±13.62	83.79±16.67
		Age(y)	43.27±8.63	36.85±10.83
	Biochemical	TG (mg/dl) (median (IQR))	165.37(101-190)	173.55(103-187)
		CHOL (mg/dl)	195.03±34.00	186.22±33.25
		HDL (mg/dl)	45.51±10.59	42.24±13.08
		LDL (mg/dl)	119.20±26.62	113.61±37.60
		FBS (mg/dl)	108.80±30.91	102.25±28.81
		INS (mg/dl)	15.51±6.08	19.47±13.31
		T. BILL (mg/d)	1.08±0.52	0.91±0.36
		D.BILL (mg/d)	0.25±0.15	0.24±0.20

The data were analyzed with two independent group t test for each characteristic, ALT: alanine aminotransferase, AST: aspartate transaminase, ALP: alkaline phosphatase, BMI: body mass index, TG: triglyceride, CHOL: total cholesterol, HDL: high density lipoprotein, LDL: low density lipoprotein, FBS: fasting blood sugar, INS: insulin, T.BILL: total bilirubin, D.BILL: direct bilirubin

**Table 2:** Serum enzymatic parameters of the study groups after the trial (Mean±SD)

Variables	Curcumin N=27		Placebo N=29		p-Value			
	Before	After	Before	After	P_1_	P_2_	P_3_	P_4_
AST(IU/L)	49.93±22.73	31.72±16.86*	50.37±17.47	31.59±11.15*	0.72	0.042*	<0.01**	0.17
ALP(IU/L)	203.10±68.53	200.38±83.34	212.67±46.04	209.70±52.33	0.65	0.06	0.69	0.81
ALT(IU/L)	73.28±29.07	42.62±23.76*	74.26±29.74	48.44±27.32	0.52	0.031*	<0.01**	0.041*

ALT: alanine aminotransferase, AST: aspartate transaminase, ALP: alkaline phosphatase. P_1_ is the p value of comparing nano-curcumin with placebo before intervention. P_2_ is the p value of comparing nano-curcumin with placebo after intervention. P_3_ is the p value of comparing data in nano-curcumin group between before and after using pair T test. P_4_ is the p value of comparing data in placebo group between before and after using pair T test.

## Discussion

The finding of this present study showed that nano-micelle curcumin reduces hepatic enzymes (ALT, and AST) serum levels in NAFLD patients. Also, a change in AST serum level in the placebo group was shown in this study which was brought about by lifestyle modifications.

ALT and AST are aggregated in the cytosol of hepatocytes. ALT is mainly expressed in hepatic cells but it also exists in muscles, adipose tissues, intestines, colon, prostate, and brain that have a much lower concentration than the liver (Liu et al., 2014 ▶). AST is not as specific as ALT to hepatocytes. When liver destruction occurs, liver enzymes (mainly ALT) are released from injured liver cells; therefore, this causes a significant elevation in serum ALT activity (Liu et al., 2014 ▶). A semi-quantitative ultra-sonographic fatty liver indicator has a close association with NAFLD (Ballestri et al., 2017 ▶). 

Based on the literature, similar previous studies have utilized different curcumin dosages. Rahmani et al. used 70 mg/day curcumin dose which led to a reduction in body mass index (BMI), liver fat content, serum lipid profile such as total cholesterol (TC), low-density lipoprotein (LDL), triglycerides (TG), as well as serum hepatic enzymes (ALT and AST), and glycated hemoglobin (Rahmani et al., 2016 ▶); a couple of studies which administered 200 mg/day curcumin led to lowering BMI, waist circumference, and serum level of uric acid, lipid profile (TC, TG, LDL-cholesterol, and non-HDL-cholesterol), and AST and ALT enzymes. However, high-density lipoprotein (HDL)-cholesterol and fasting blood sugar (FBS) did not change, and serum hepatic enzymes (ALT and AST) elevated (Panahi et al., 2016 ▶; Hosseini et al., 2018 ▶). Conversely, the current study observed a significant decrease in serum ALT and AST level in the curcumin group and a meaningful reduction of AST serum concentration in the placebo group; additionally, no meaningful association was discovered between curcumin and BMI, demographic variables, serum level of lipid profile, FBS, insulin, total bilirubin, direct bilirubin, or ALP. This negative result (for ALP changes) may be caused by recommendation of low-calorie consumption and more physical exertion in both groups and participants over attention to this advice; interestingly, this possible reason is in agreement with significant lowering AST serum level in the placebo group in the present study. Moreover, lack of adequate AST concentration in hepatocytes might lead to considerable decrease of AST serum level in the placebo group. Different doses of curcumin exert different effects on NAFLD. Thus, more than one factor can influence the results cooperatively. 

In our study, it is emphasized that combination of nano-micelle curcumin with modification of life style and eating habits is the best and most effective protocol in NAFLD management. Our investigation must be interpreted with caution because, among all examined variables (anthropometric, demographic, and biochemical), just serum ALT concentration showed a significant decrease in the curcumin group; therefore, the information involves novel insight into possible shared functional features among ALT and curcumin targets. Furthermore, we can conclude that serum ALT level is one of the most dependent factors in NAFLD patients, and so we should consider ALT blood concentration in the diagnosis and prognosis of NAFLD condition. 

Prior studies have noted the importance of hepatic enzymes (ALT and AST) elevation in association with NAFLD. One study by Liu et al. stated a non-linear correlation between the histologic severity of NAFLD and the extent of ALT elevation (Liu et al., 2014 ▶). Schindhelm et al. found that ALT elevation is related to associate hepatic steatosis diseases such as atherosclerosis, type 2 diabetes mellitus, and metabolic syndrome (Schindhelm et al., 2006 ▶). Weiß claimed that abnormally high serum AST and ALT enzymes level can have diagnostic potential in steatosis patients (Weiß et al., 2014 ▶). In another major study, Rinella et al. pointed out that non-alcoholic steatohepatitis (NASH) patients with high ALT serum levels had more associate to insulin resistance and intrahepatic fat content (Rinella, 2015 ▶). In another study, Yki-Järvinen demonstrated that liver fibrosis progression was linked to lowering hepatic ALT activity and increasing AST/ALT ratio (Yki-Järvinen, 2016 ▶). With regard to the mentioned evidence and our observation, nano-curcumin has a significant effect in improving NAFLD signs and other associated diseases such as atherosclerosis, metabolic syndrome, and hepatic fibrosis through reducing ALT and AST serum levels. This finding broadly supports the work of other previous studies in this area treatment of NAFLD with curcumin. Experimental studies have shown that curcumin supplementations improve NAFLD signs and its related diseases such as metabolic syndrome and diabetes mellitus type 2 (Inzaugarat et al., 2017 ▶; Cicero et al., 2018 ▶; Cunningham et al., 2018 ▶). Various mechanisms have been demonstrated hepatoprotective effects of curcumin such as inhibiting the NF-κB pathway of activated B cells which promotes liver injury (Rivera-Espinoza and Muriel, 2009 ▶) and targeting AMP-activated protein kinase to inhibit hepatic lipogenesis in order to decrease fat accumulation in the liver (Um et al., 2013 ▶). Moreover, in a high-fat diet-induced NAFLD/nonalcoholic steatohepatitis mouse model study, oral administration of curcumin reduced intrahepatic CD4+ cell accumulation and the underlying linoleic acid- and leptin-induced pro-inflammatory and pro-oxidant effects on mouse liver macrophages (Inzaugarat et al., 2017 ▶). Furthermore, a high-fat and high-fructose diet-induced NAFLD mice model study suggested LXRα pathway might be a novel therapeutic target of curcumin in NAFLD patients (Larasati et al., 2018 ▶). As the oral bioavailability of curcumin is poor indeed in mice and humans, curcumin’s solubility in water is about 0.0004 mg/ml at pH 7.3 (Yallapu et al., 2012 ▶). An efficient method for best bioavailability should be established to overcome the unstirred water layer which is a water layer on the surface of gastrointestinal epithelial cells to help best absorption (Rahimi et al., 2016 ▶; Hatamipour et al., 2019 ▶). Different adjuvants have been utilized like amorphous solid dispersion (Rahmani et al., 2016 ▶) and phytosomal chemicals (Panahi et al., 2016 ▶; Rahimi et al., 2016 ▶; Hosseini et al., 2018 ▶) to enhance curcumin effectiveness in recent similar studies. In the current research nano-micelles containing curcumin (SinaCurcumin^®^) were used which have a higher efficacy value of delivery unlike nano-crystals and other conjugates (Rahimi et al., 2016 ▶). Other studies used curcumin that contained high gross substances which cause interruption through treatment procedures and goals of their analysis for NAFLD (Panahi et al., 2016 ▶; Hosseini et al., 2018 ▶). Nano-micelles curcumin has very good bioavailability (the curcumin Cmax of approximately 2540.62 nmol/l was reached after 30 min). Rahimi et al. had described all types of novel drug delivery for curcumin in recent years, and overall nano-micelles was considered to be a very effective method (Rahimi et al., 2016 ▶; Hatamipour et al., 2019 ▶).

Serum aminotransferase levels and imaging tests such as ultrasound, CT, and MR do not reliably assess steatohepatitis and fibrosis in patients with NAFLD.

Curcumin nano-micelle used in our study was almost 100% pure and lacked gross substances, making our results to be more trustable. This study is subject to some limitations. First, the study was conducted with one dosage of curcumin like former studies. According to the chemical molecule of curcumin has been shown bioavailability due to be time and dose-dependent. Therefore, more studies with bigger sample size still need to be carried out in order to find the optimum time and dosage of intervention (Mansour-Ghanaei et al., 2019 ▶). Another limitation of this study was that the participants were assessed with ultrasonography for the diagnosis of NAFLD rather than the hepatic biopsy and histological test due to their invasive nature. It is also recommended to test gamma-glutamyl transferase and spleen size that could serve as indices of NAFLD progression which were not assessed in this study. In addition, future works should consider the relationship between ALT and curcumin serum targets level. Although curcumin has been recognized as a safe medication with no significant complications, studies have mentioned some of its interactions with midazolam, tacrolimus, cyclophosphamide, and celiprolol (Mansour-Ghanaei et al., 2019 ▶) that should be taken into account in further studies. 

In conclusion, our findings showed that oral administration of 160 mg/day curcumin for 60 days ameliorates conditions of NAFLD. Moreover, it significantly lowers the serum concentrations of ALT and AST. The treatment methods for NAFLD are now limited to lifestyle modification and physical activity, and no certain cure has been proposed. Our study along with other similar research will help to find a pharmaceutical approach toward this problem. 

## Conflicts of interest

The authors have declared that there is no conflict of interest.
